# The malaria parasite chaperonin containing TCP-1 (CCT) complex: Data integration with other CCT proteomes

**DOI:** 10.3389/fmolb.2022.1057232

**Published:** 2022-12-08

**Authors:** Mark D. Wilkinson, Josie L. Ferreira, Morgan Beeby, Jake Baum, Keith R. Willison

**Affiliations:** ^1^ Department of Life Sciences, Imperial College London, London, United Kingdom; ^2^ School of Biomedical Sciences, University of New South Wales, Kensington, NSW, Australia; ^3^ Department of Chemistry, Molecular Sciences Research Hub, Imperial College London, London, United Kingdom

**Keywords:** chaperonin, *P. falciparum*, autophagy, WD40 propellors, cytoskeleton, protein coacervate, natively disordered protein

## Abstract

The multi-subunit chaperonin containing TCP-1 (CCT) is an essential molecular chaperone that functions in the folding of key cellular proteins. This paper reviews the interactome of the eukaryotic chaperonin CCT and its primary clients, the ubiquitous cytoskeletal proteins, actin and tubulin. CCT interacts with other nascent proteins, especially the WD40 propeller proteins, and also assists in the assembly of several protein complexes. A new proteomic dataset is presented for CCT purified from the human malarial parasite, *P. falciparum* (PfCCT). The CCT8 subunit gene was C-terminally FLAG-tagged using Selection Linked Integration (SLI) and CCT complexes were extracted from infected human erythrocyte cultures synchronized for maximum expression levels of CCT at the trophozoite stage of the parasite’s asexual life cycle. We analyze the new PfCCT proteome and incorporate it into our existing model of the CCT system, supported by accumulated data from biochemical and cell biological experiments in many eukaryotic species. Together with measurements of CCT mRNA, CCT protein subunit copy number and the post-translational and chemical modifications of the CCT subunits themselves, a cumulative picture is emerging of an essential molecular chaperone system sitting at the heart of eukaryotic cell growth control and cell cycle regulation.

## Introduction

The multi-subunit chaperonin containing TCP-1 (CCT) complex (also named TRiC, the TCP-1 Ring Complex) is a 1 MDa ATPase which functions in cytosolic protein folding and protein assembly reactions in all eukaryotes (Willison, 2018a). Upon binding and hydrolysis of ATP, CCT encapsulates and folds non-native proteins. CCT also facilitates assembly of many proteins with their respective partners using, at present, poorly understood mechanisms. CCT is constructed from two identical, but opposed, rings made by eight independent subunits, encoded by eight homologous genes, and positioned in each ring in a precise arrangement. Each CCT subunit consists of three domains: the equatorial domain harboring the ATP binding site, an intermediate domain, and the apical domain involved in substrate binding. The specific sequence differences between the eight CCT subunits are located mainly in their apical domains which provide the substrate binding specificity of each subunit. All eight CCT genes are essential in yeast and even minor perturbations of any of their ATP-binding site motifs and ATP-hydrolysis kinetics result in loss of viability and severe growth defects which include abnormally large cell sizes and defective cell shapes (Amit et al., 2010). Previous work (Willison, 2018a) has reviewed the CCT structure and allosteric mechanism and its role in normal biological processes and in disease, developing a model for the role of CCT in cell cycle and growth control. One key element of the model is the connection between actin and tubulin protein folding flux with protein modules involved in APC ubiquitination, the TOR pathway and others (Willison, 2018b).

Advances in protein mass spectrometry and proteomics over the past 25 years has led to an almost complete description of the copy number distribution of all proteins in yeast (Ho et al., 2018). The integration of protein abundance data with other global-omics data such as genetic (Costanzo et al., 2010) and chemical screens (Hillenmeyer et al., 2008), post-translational modifications and mRNA transcriptomics provides a steady-state wiring diagram (Costanzo et al., 2016) of the protein composition and protein-protein interactions in yeast. Over the coming years, time-resolved measurements of yeast cell signaling, and cell morphogenesis processes will be attached to the wiring diagrams. These approaches, pioneered in yeast, are now generating comparable datasets in model eukaryotic organisms and humans. Bioinformaticians have constructed large-scale databases, such as the Saccharomyces Genome Database (SGD) and Omics Discovery Index (OmicsDI), which can be interrogated precisely by investigators to locate the available information about their system of interest. Given that the CCT proteome has been reviewed extensively elsewhere, the purpose of this article is to guide readers towards informative, accurate and well curated datasets and associated experiments and, at a more granular level, highlight aspects of CCT function and behavior which are biologically significant and lie at the core of the CCT systems network (Dekker et al., 2008; Aswathy et al., 2016; Rizzolo et al., 2017). Extending the field, here we also bring into focus the role of CCT in the divergent and less well-studied protozoan parasite, *Plasmodium*, the causative agent of human malaria.

## Materials and methods

### Tagging the PfCCT complex

Parasites were transfected with a vector designed to have a CCT8-FLAG tag integrated using selection linked integration (SLI) following established protocols (Birnbaum et al., 2017). Briefly, parasites were transfected with vector and selected under 4 nM WR99210 for a period of 1–4 weeks. Wild-type parasites are unable to grow whereas parasites containing the plasmid (containing a hDHFR positive selection cassette) are able to grow. Once parasites recovered from transfection and grew normally, they were subjected to G418 drug pressure (selecting for integration of a second Neomycin-resistance selection cassette) and parasites that had successfully integrated the DNA from the plasmid (FLAG-tagged CCT8) grew. PCR assays tested for the presence of the wild-type gene (no tag), the plasmid (no protein product) and successful integration. Successful integrants were screened by western blot and immunofluorescence assays using an anti-FLAG antibody, which recognizes the tagged protein. Parasites did not grow when transfected with five of the eight subunit constructs and one of the subunits (CCT4/δ) was identified by western blot but still retained significant wild type locus and was therefore discarded. Two of the subunits (CCT1/α and CCT8/θ) were successfully tagged and retained no wild-type locus when probed by PCR. CCT8/θ had the highest ex-pression levels by western blot and a better level of integration (as shown by PCR) and was selected as the parasite line for further analysis of the PfCCT complex.

### Purification of the CCT8-FLAG complex from *P. falciparum*

30 ml of the CCT8-FLAG parasite line (4% haematocrit) intended for large scale culture was sorbitol synchronized on day 1 to 0.5% parasitemia, day 3 to 0.8% parasitemia and day 5 to a maximum parasitemia of 9%. Following synchronisation on day 5, parasites were diluted into 8-12 flasks to a final volume of 100 ml at 2% haematocrit, 0.5% parasitemia per flask, with each 100 ml representing a final volume of 300 ml at 4% haematocrit. On day 8, parasites were smeared to ensure they were late stage and synchronous to a parasitemia of no higher than 6%. Parasites were then transferred to 400 ml flasks and supplemented with 250 ml pre-warmed complete media and the haematocrit was adjusted to 2%. On day 9, parasites were rested for 1 h at room temperature to allow erythrocytes to settle before a complete media change.

Day 10 parasites were Giemsa smeared to ensure parasites were at the correct stage (late trophozoite/early schizont). The flasks were rested for 1 h at room temperature to allow RBCs to settle before aspirating media off the surface. The RBCs of each flask were then transferred to a 50 ml falcon (one per flask) and pre-warmed complete media was used to rinse any remaining RBCs from the flask. The RBCs were pelleted using centrifugation (room temperature, 800 G, brake 3). The blood pellet was resuspended in five times its volume in saponin buffer (0.075% saponin in parasite wash buffer (PWB): 30 mM HEPES pH 8.0, 300 mM NaCl, 2.5% v/v glycerol, 2 mM EDTA, 4 mM DTT) and resuspended for 5 min at room temperature before being inverted and incubated for another 5 min. The parasites were pelleted using centrifugation (4°C, 2,800 G, 10 min) and kept on ice from here onwards. The parasites were then resuspended using 4°C PWB and pelleted as before and combined with one other 50 ml falcon on each wash until only one 50 ml falcon containing the total parasite pellet remained (typically 5–7 ml). Parasites were lysed using nitrogen cavitation. The parasite pellet was resuspended to double the pellet volume in parasite wash buffer supplemented with an EGTA-free protease inhibitor cocktail (Roche). This was transferred into a pre-chilled nitrogen cavitation chamber (Parr Instrument Company) and incubated on ice at 1500 psi for 20 min before being agitated and the pressure topped up to 1500 psi and left for another 20 min on ice. The parasite lysate was carefully released from the chamber into a chilled 50 ml falcon and clarified by centrifugation; 18,000 G for 30 min at 4°C. The clarified lysate was then incubated with 100 μl of anti-FLAG resin (Sigma) at 4°C overnight, pelleted and washed twice in CCT purification buffer (30 mM HEPES pH 8.0, 300 mM NaCl, 2.5% glycerol, 2 mM EDTA, 4 mM DTT and one EDTA-free protease tablet). The resin-bound fraction was then incubated with FLAG peptide (50 μg/ml) in CCT purification buffer at 4°C for 1 h. The resin was pelleted by centrifugation at 1,000 G for 10 min at 4°C. The anti-FLAG resin elution was then applied to a 10–40% sucrose gradient supplemented with 90 mM KCl, 50 mM HEPES pH 7.2 and one EGTA-free protease inhibitor (Roche) and centrifuged at 25,000 RPM (SW41i) overnight at 4°C. Fractions containing the CCT complex, confirmed by SDS-PAGE and Western blot, were pooled and flash frozen in liquid nitrogen.

### Negative stain electron microscopy of the PfCCT complex

The purity and integrity of the sample was assessed by negative stain electron microscopy. 3 ul of protein at 1 mg/ml from the 20% sucrose peak fraction was applied to glow-discharged continuous-carbon grids (Taab Laboratory Equipment Ltd.), stained with 2% uranyl acetate and stored at room temperature prior to imaging. Negative stain micrographs were collected on a Tecnai T12 TWIN (Lab6) using a TVIPS F216 CCD camera. Images were recorded with a pixel size of 2.586 Å. Processing of single particles was done within Scipion 1.2, 900 particles were picked manually and then a further ∼9000 particles were picked using Xmipp3. 2D classification was done using Relion 2.1.

### FLAG-tag affinity purification and MS analysis

Parasite lines CCT-theta-FLAG and RNAP2-FLAG (control, unpublished data) were grown to 1 L volume and separately harvested and lysed up until incubation with anti-FLAG resin. Each culture was incubated separately with 200 µl anti-FLAG resin overnight at 4°C. Each FLAG resin-bound sample was then washed in a wash buffer containing 30 mM HEPES pH 8.0, 300 mM NaCl, 2.5% glycerol, 2 mM EDTA, 4 mM DTT and one EDTA-free protease tablet. The bound fractions were then sent to the University of Bristol Proteomics Facility for Tandem Mass Tagging (TMT), providing a quantitative readout of sample from the beads. All spectra were acquired using an Orbitrap Fusion Lumos mass spectrometer controlled by Xcalibur 3.0 software (Thermo Scientific) and operated in data dependent acquisition mode. The raw data files were processed using Proteome Discoverer software v2.1 (Thermo Scientific) and searched against the UniProt *P. falciparum* database (downloaded xxx 2019; xxx sequences), an inhouse “common contaminants” database. Peptide precursor mass tolerance was set at 10 ppm, and MS/MS tolerance was set at 0.6 Da. Search criteria included oxidation of methionine (+15.995 Da), acetylation of the protein N-terminus (+42.011 Da) and methionine loss plus acetylation of the protein N terminus (−89.03 Da) as variable modifications and carbamidomethylation of cysteine (+57.021 Da) as a fixed modification.

## Results

### Tagging and purification of PfCCT

**FIGURE 1:**
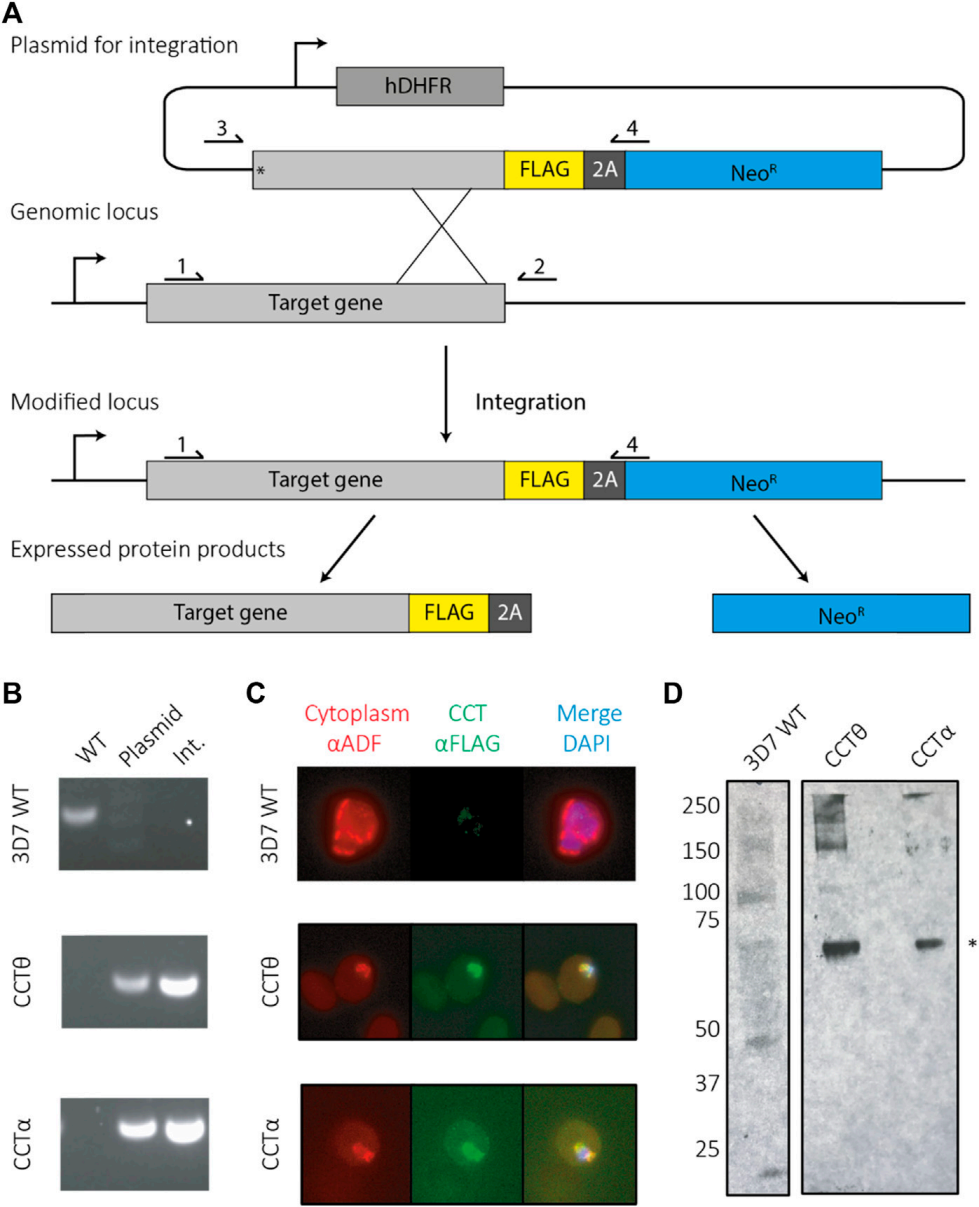
Tagging PfCCT using selection linked integration (SLI). **(A)** A schematic for tagging using SLI. Parasites are first selected under the hDHFR cassette for successful transfection of the plasmid, which integrates to the genomic locus using homology to the target gene. Successful integrants then contain a tag (here a FLAG tag). When expressed the ribosome skips over the 2A skip peptide and produces the tagged gene of interest as well as a neomycin resistance protein, enabling parasite to grow under G418 drug pressure. **(B)** Primers sites are labelled 1–4 for checking parasites by PCR for successful integration events. Wild type (WT) parasites are identified using primers 1 and 2. Parasites containing plasmid can be identified using primers 3 and 4 and successful integrants confirmed using primers 1 and 4. **(C)** Immunofluorescence assays show the tagged CCT complex colocalises with cytoplasmic ADF. **(D)** An anti-FLAG western shows both CCT8/CCTθ and CCT1/CCTα are present at their expected molecular weights (asterisk—65 kDa).

CCT is essential for asexual development in *P. falciparum* but only over-expression methods have been used to produce CCT with tagged subunits (Spillman et al., 2017). We sought to endogenously tag the CCT complex in the parasite for purification using the Selection Linked Integration (SLI) protocol (Birnbaum et al., 2017) which has been established as the quickest and most efficient method for modifying an endogenous locus. SLI overcomes issues with inefficient homologous recombination in *P. falciparum* and allows genetic modifications within 21 days. In order to carry out SLI tagging of the CCT complex we tagged each of the 8 CCT subunit genes separately to find which were amenable to receiving a 3-FLAG purification tag at the C-terminus. Eight plasmids were constructed, each one containing the human dihydrofolate reductase (hDHFR) pyrimethamine (WR) resistance cassette and an 800 base pair segment homologous to the C-terminus of the respective CCT subunit. The homology regions were preceded by a stop codon to ensure there was no basal expression from the plasmid. The 3-FLAG purification tag, consisting of the DNA sequence coding the tag and a T2A skip peptide preceded the neomycin resistance gene, enabling the parasites to grow under G418 drug selection (Figure 1A). CCT1 and CCT8 genes were successfully tagged and had no wild-type locus by PCR analysis (Figure 1B) and both tagged CCT subunits were located in the cytoplasm, the expected cellular compartment, as shown by anti-FLAG antibody staining (Figure 1C). Western blot analysis shows protein signal at the expected monomer molecular weight (Figure 1D). CCT8 had the highest expression levels by western blot and a better level of integration, as shown by PCR, and was selected as the parasite line for further analysis of the PfCCT complex.

### PfCCT expression profiles

**FIGURE 2:**
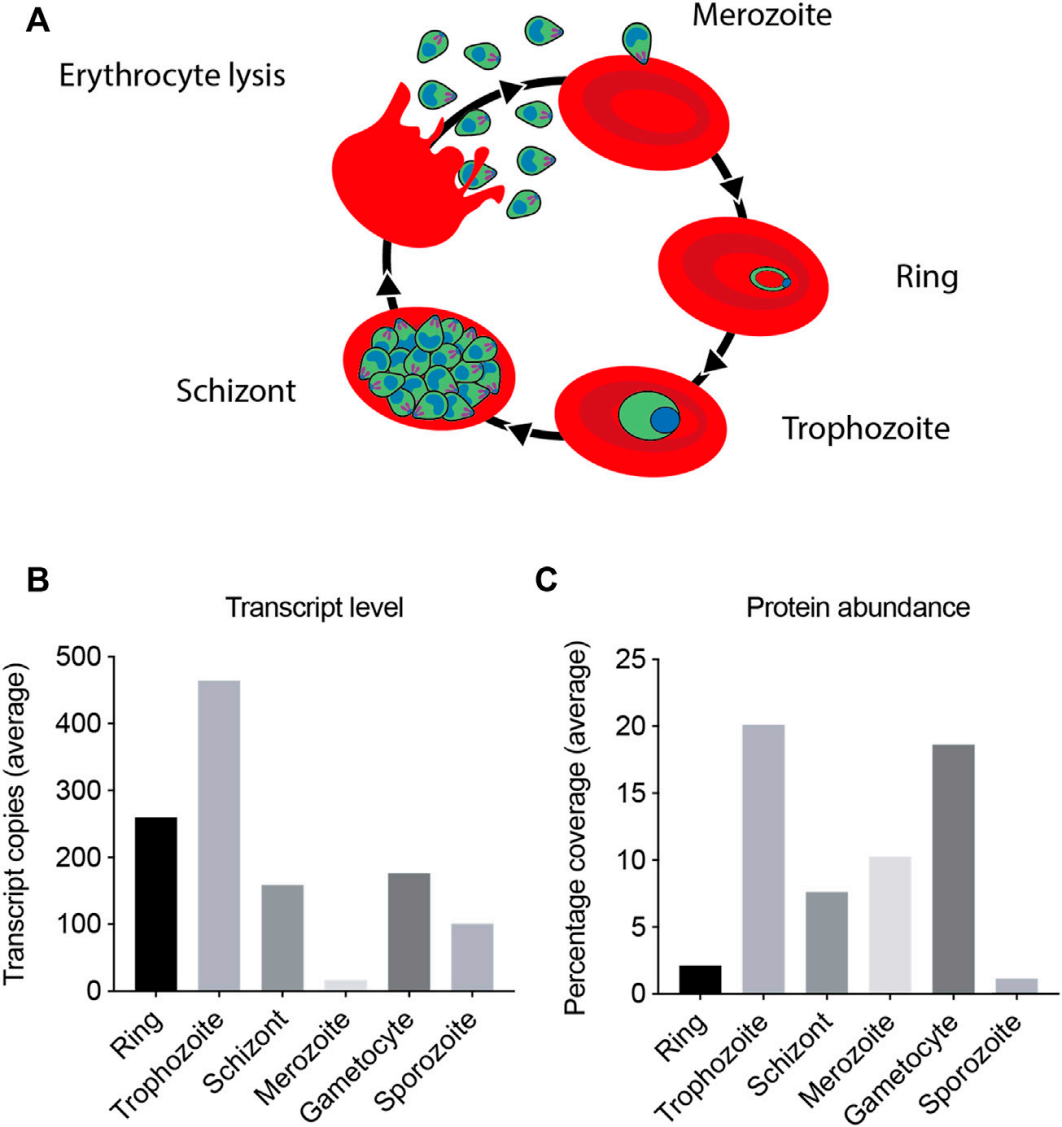
PfCCT complex mRNA transcript and protein expression levels. The PfCCT complex mRNA and protein expression levels have been monitored in a number of studies (Florens et al., 2002) (Nirmalan et al., 2004) (Bozdech et al., 2003), with maximal transcription and expression observed at the trophozoite stage. **(A)** Diagram of the asexual life cycle of *P. falciparum* in the human erythrocyte. **(B)** The transcript levels shown are the average number of RNA copies of all the eight subunits in the CCT complex, generated by microarray. **(C)** The protein expression levels shown are average percentage coverage of all the CCT subunits obtained by mass spectrometry analysis which is indicative of the CCT subunit protein abundance in the sample.

To maximize yields of the CCT complex we aimed to purify it from the asexual growth stage when the parasite is expressing the greatest amounts of the protein. Transcription and proteomic analyses were conducted in order to establish the stage at which the parasite is maximally expressing the CCT complex (Figure 2) (Florens et al., 2002; Bozdech et al., 2003; Nirmalan et al., 2004). Combined, these data show that the maximum transcript levels and expression levels are in the trophozoite stage of the asexual life cycle. The trophozoite stage is the feeding and growth stage, which necessitates increased protein production by the parasite. The trophozoite stage is also physically one of the largest stages of the parasite having greatest biomass.

### PfCCT purification and image analysis

**FIGURE 3:**
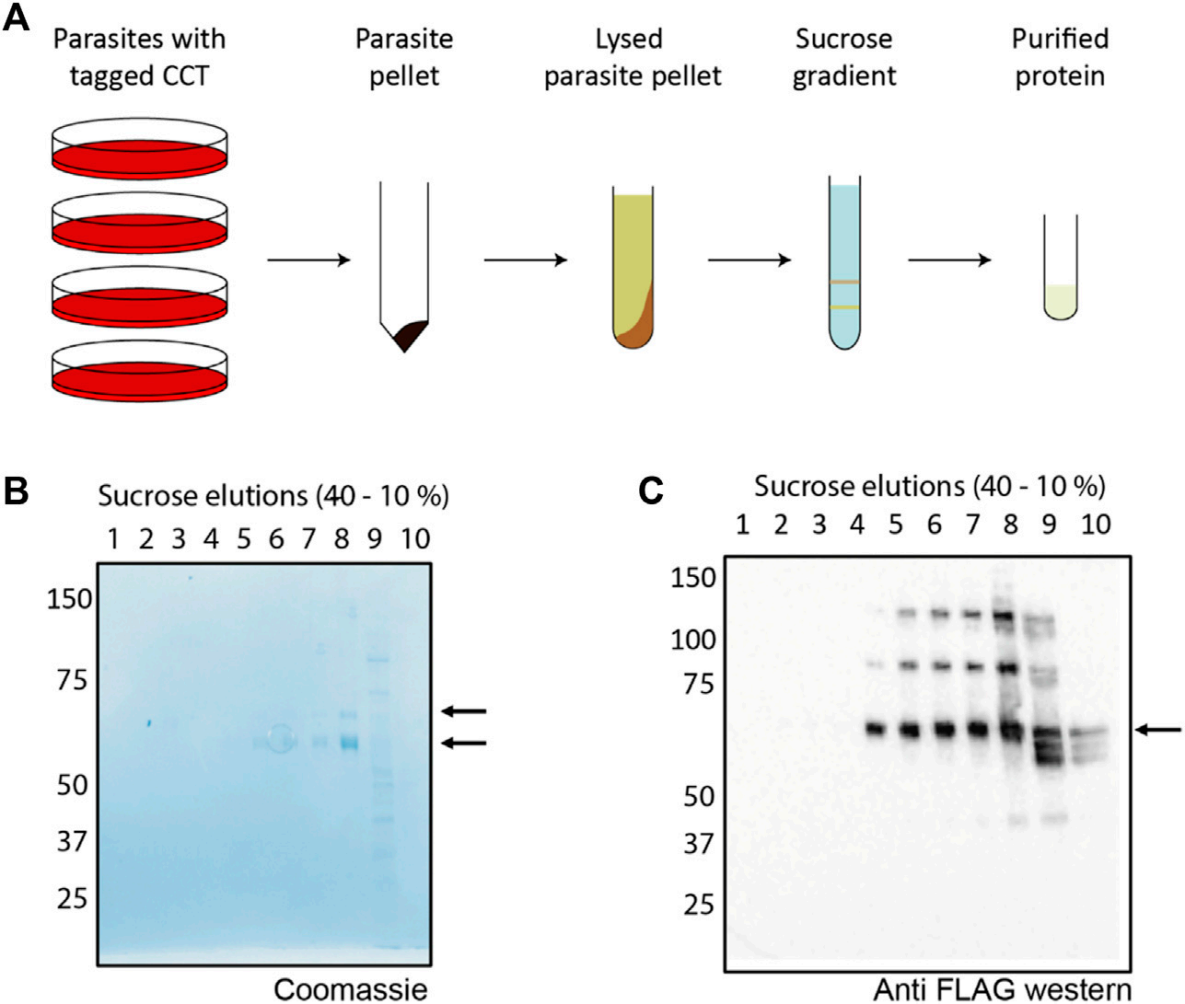
Purification scheme and analysis of PfCCT **(A)** A general schematic for purification of the CCT complex from *P. falciparum*. Parasites containing an endogenous tag for PfCCT purification were grown, isolated using saponin lysis and lysed by nitrogen cavitation. The parasite lysate was then incubated with anti-FLAG resin before eluting the bound fraction for application to a 10%–40% sucrose gradient. **(B)** A Coomassie stained SDS-PAGE gel shows the elution peak from the anti-FLAG resin contains the tagged CCT8 subunit (top arrow, ∼65 kDa) and the “CCT box” containing the rest of the seven subunits (∼60 kDa, bottom arrow). **(C)** The elution peak is applied to a 10%–40% sucrose gradient (Lewis et al., 1992). Gradient fractions are applied to SDS-PAGE gel and Western blotted with anti-FLAG antibody to detect shows the presence of the FLAG-tagged CCT8 subunit.

The parasite lysate soluble fraction (Figure 3A) was first incubated with a resin conjugated to an anti-FLAG antibody, which binds to the 3x-FLAG tag on the C terminus of CCT8. The resin-bound fraction was eluted by incubation with excess FLAG peptide which displaced the PfCCT complex into the flow through. SDS-gel electrophoresis shows the tagged CCT8 subunit, ∼ 5 kDa larger than the other seven subunits migrating closely together ∼60 kDa (Figure 3B). The combined elution fraction volume was small (1 ml) and the quantity of protein was low so we used a sucrose gradient, which does not dilute the protein and has been used for CCT purification from mouse and yeast (Lewis et al., 1992; Dekker et al., 2011). A gradient of 10%–40% sucrose w/v causes CCT holo-complexes to sediment at ∼20% sucrose density (Figure 3C). Western blot analysis of the sucrose gradient fractions with anti-FLAG antibody (Figure 3C) identified multiple biochemical species of CCT8-FLAG in addition to the holo-CCT complex at expected 20% sucrose density (Lewis et al., 1992). Spillman et al. (2017) also observed monomeric forms and micro-complexes (Liou and Willison, 1997) for all PfCCT subunits examined and determined that the micro-complexes observed for their tagged CCT8 (TRiC-θ) subunit were different to those of other sub-units. Nevertheless, the material found in fraction 8 of our 10%–40% sucrose gradient, which is at the expected sucrose concentration of around 15%–20% w/v sucrose was of suitable purity and concentration for further characterization by negative stain electron microscopy.

**FIGURE 4:**
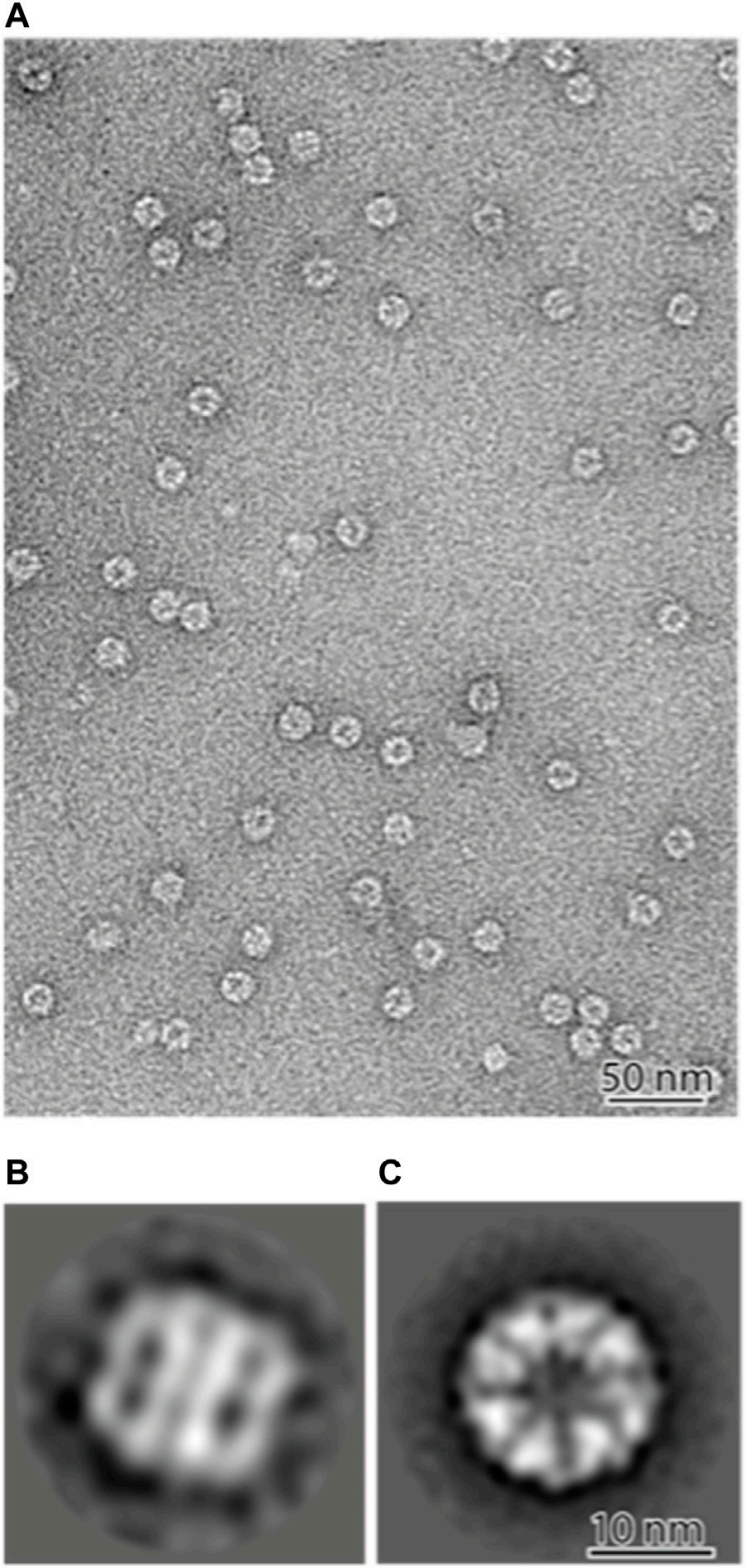
Negative stain electron microscopy of the PfCCT complex **(A)** ×100000 nominal magnification. The majority of the complexes are vertical on the grid and therefore show “top-down” views. **(B,C)** 2D classifications of 9025 particles shows the majority of the particles are top-down views **(C)** with a minority of side views **(B)**. The PfCCT complex measures 170 Å in diameter and 180 Å in height, with each subunit around 60 Å in width.

2D classification showed that most of the views were top-down, with some side-on views (Figure 4A). The resolution was sufficient to image the 8 individual subunits in two rings, showing that the PfCCT complex forms the expected hetero-hexadecamer. The PfCCT complex has a diameter of around 170 Å and a height of 180 Å. Each subunit is approximately 60 Å in size. This correlates well with the crystal structure of the CCT complex from yeast (Dekker et al., 2011) which measures around 150 Å by 160 Å in size. The marginal increase in size might be attributed to the negative staining process, which expands the apparent dimensions of the protein complex. We note though that PfCCT shows significant population of higher density fractions in the sucrose gradient, 20%–30% (Figure 3C), and it is possible that these higher density CCT species are expanded because they are carrying large client(s) and assembly complexes.

### Mass spectrometry of PfCCT

We endeavoured to extract CCT complex directly from parasite lysate, which in turn results in co-extraction of its binding partners of the complex as we previously accomplished for yeast CCT (Dekker et al., 2008). In order to identify the CCT interactome of the complex by mass spectrometry we compared the soluble fraction with the anti-FLAG resin bound material. As a negative control, a parasite line was used which contained a FLAG-tagged RNA polymerase complex that localizes to the nucleus (F. Fisher, unpublished data) and whose subunits are unlikely to have overlap with the CCT interactome. Both samples bound to the resin were washed to remove any weak non-specific interactions before undergoing Tandem Mass Tag labelling and mass spectrometry.

**FIGURE 5:**
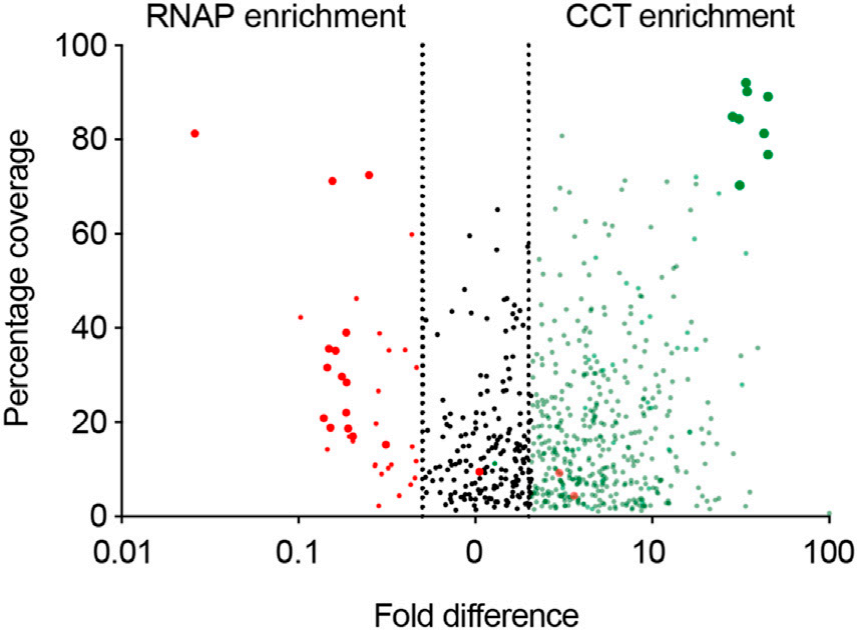
The CCT interactome volcano plot. The CCT interactome. A graph showing the difference in proteins pulled down using RNA polymerase (red) and the CCT complex (green) against percentage coverage, as an indicator of abundance. Proteins between the two dashed lines were discarded, as they were not significantly enriched in either sample (i.e., proteins with less than two-fold enrichment in either sample). RNA polymerase subunits (big red) and CCT subunits (big green) are highlighted.

**FIGURE 6:**
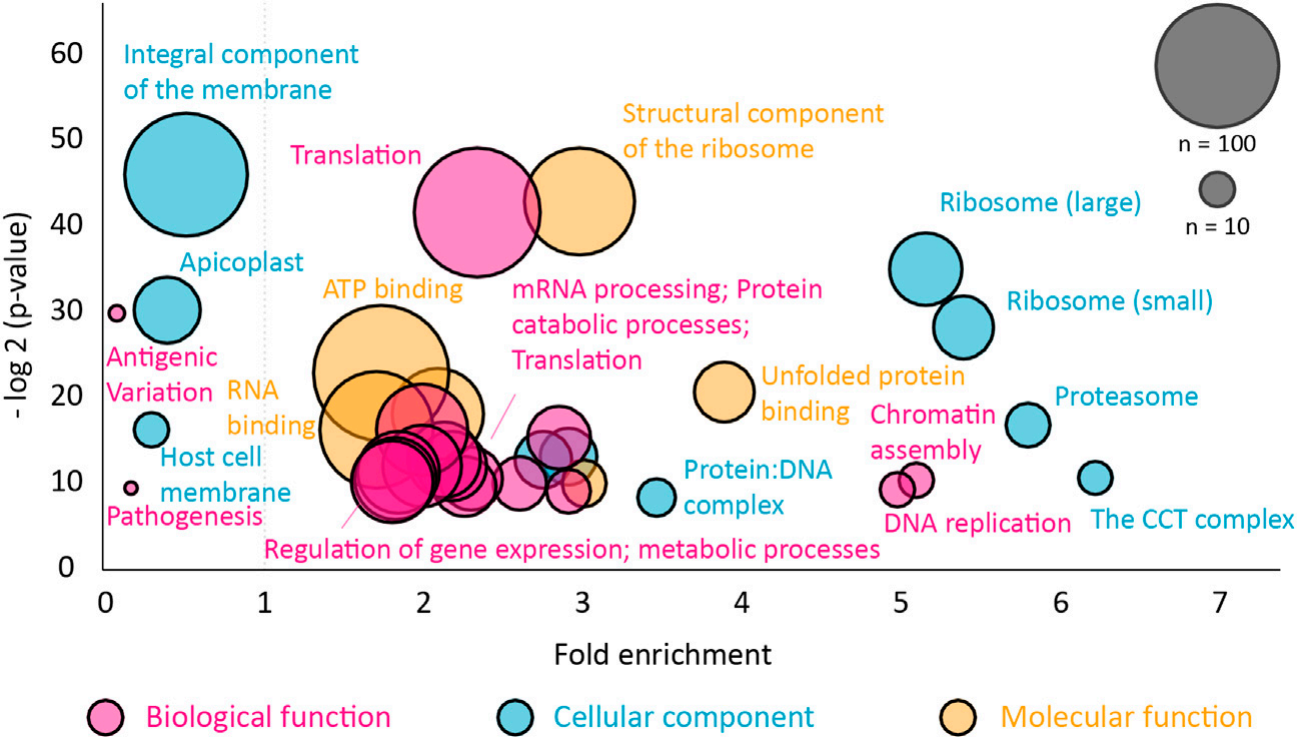
Gene ontology analysis of the PfCCT interactome. The Gene Ontology (GO) enrichment analysis was performed using the GO server PANTHER for proteins that were more than two-fold enriched compared to the RNA polymerase pulldown. The full list with gene identifiers is provided in Supplementary Data S1. Node size corresponds to the number of proteins found in the class, color represents GO class and enrichment is relative to the *P. falciparum* proteome. The probability (*y*-axis) is the probability of seeing at least x number of genes out of the total n genes in the list annotated to a particular GO term. Any class found below 1× fold enrichment is less than expected relative to the *P. falciparum* proteome.

The pulldown identified 896 proteins that were more than two-fold abundant in the CCT pulldown compared to the RNA polymerase pulldown (Figure 5). It was encouraging to see that the highest sequence coverage was for the 8 CCT subunits, highlighted by largest green points in Figure 5 and shown in Table 1. Many known CCT interaction partners were widely covered and highly abundant including actin, tubulin, and proteins with expected WD40 motifs (Table 1 and Supplementary Data S1). The proteins identified by this technique represent 15% of the total proteome in *P. falciparum*, which has around 5,400 protein coding genes. This wide-reaching network of the PfCCT complex with almost 1,000 proteins highlights the importance of the complex in parasite growth and survival. To further explore the PfCCT interactome we carried out Gene Ontology (GO) analysis, which groups genes or proteins by their function within the cell, to probe for biological function, cellular components, and molecular function (Figure 6). Of the 896 proteins, 19 were unmapped in the *P. falciparum* gene ontology groups and therefore the analysis was carried out on 877 proteins. Gene Ontology enrichment analysis groups genes or proteins by their function within the cell. This grouping is carried out on both the whole proteome and the sample. Enrichment analysis then calculates the probability of the protein and/or function being randomly identified in the sample. For example, the *P. falciparum* proteome has 5,441 total proteins, of which 59 are in the “protein folding biological function” group. A random pulldown of 877 proteins would expect to have 9.51 proteins related to protein folding (i.e., (59/5441) × 877 = 9.51). In the CCT pulldown sample, there were 27 in this protein folding biological function, which represents a 2.84-fold increase (the actual ÷ expected: 27÷9.51) and a calculated *p*-value of 2.39E-05, calculated using Fisher’s exact test. This is done for every protein identified in the sample using the online server PANTHER (Mi et al., 2019). The GO analysis of the proteins identified in this pulldown highlights the range of pathways that the CCT complex interacts with within the *Plasmodium* cell, from translation (ribosomes, mRNA processing, translation) through to DNA replication (chromatin assembly) and protein folding (unfolded protein binding) (Figure 6). The GO analysis also suggests the complex has less involvement with proteins involved in pathogenesis and host-cell interaction since fewer of these processes represented in the sample than expected (Figure 6) although, amongst the top 150 hits, 5 *Plasmodium* specific antigen proteins were detected bound to PfCCT: erythrocyte surface antigen, 101 kDa malarial antigen, liver stage antigen, antigen 332, and ring exported protein. It should be noted that sample preparation actively removes the erythrocyte membrane and other insoluble membrane components and therefore biases for intracellular proteins and not those that are expressed externally and involved in pathogenesis. Nonetheless, these data support the use of the CCT8/θ tagged CCT complex for defining its interactome in trophozoite cytosol.

**TABLE 1:** Selected hits from the PfCCT proteome.

Protein name	UNIPROT	Coverage %
CCT1	Q8II43	81.3
CCT2	O97247	90.2
CCT3	Q8I5C4	89.1
CCT4	C0H5I7	84.9
CCT5	O97282	70.3
CCT6	C6KST5	84.3
CCT7	O77323	92
CCT8	O96220	76.8
Actin I	A0A144A1R5	72.1
Actin II	A0A144A3N9	55.9
Actin-like	Q8I450	28
Actin-related	Q8IBQ9	13.9
Tubulin α-chain Chr 9	Q6ZLZ9	60
Tubulin α-chain Chr 4	Q8IFP3	48.4
Tubulin β-chain	A0A143ZWL7	68.5
Tubulin γ-chain	Q8IAN7	42.5
Tubulin γ-chain complex	Q8I2I3	3.6
Phosducin-like 1	Q8I3U4	32.2
Phosducin-like 2	Q8IJX5	35.8
Phosducin-like 3	A0A143ZZR4	39
WD40 CAF1	Q8IE52	35.8
WD40 CAF1 complex	Q8I5D0	3
WD40 U3snoRNA	Q8ILZ2	41.2
WD40 NOP10	Q8IAL3	4.4
WD40 mRNA splicing	Q8IL37	49.6
WD40 mRNA splicing	Q8IJZ5	35.6
WD40 mRNA splicing	O97292	1.6
WD40 mRNA splicing	C0H5G7	50.8
WD40 Coatomer α	C6KSR5	17.2
WD40 Coatomer β	Q8I390	10.5
WD40 novel	Q8IKN9	7.5
WD40 novel	Q8IHX6	2.1
WD40 dsDNA break	Q8IJ73	2.5
WD40 Fe-S assembly	Q8I5V5	22.3

## Discussion

Cataloguing the endogenous cellular proteins that interact with CCT has used *in vivo* and *in vitro* labelling and 2D-PAGE display in mammalian cell cultures (Grantham et al., 2006; Yam et al., 2008) and a host of individual CCT-protein or gene interactions have been described which suggest that hundreds of proteins can interact with CCT (Willison, 2018a; Willison, 2018b). It is an ongoing task to establish the interaction behaviors of the proteins that bind to CCT. Are they folding substrates or components of protein complex assemblies or substrates for post translational modification or targets for kinetic partitioning? The most comprehensive description of the CCT proteome in a single cell type has been established in yeast (Dekker et al., 2008; Rizzolo et al., 2017) and has identified a ‘core’ CCT proteome (Willison, 2018b). In this discussion, we review some of the interaction networks and compare with the yeast datasets and we also attempt to place the results of the *Plasmodium* CCT interactome into the general context. We find conserved similarities with yeast but also identify novel CCT binding proteins of unknown function in *Plasmodium*. These hits will hopefully guide future experiments and further understanding of the molecular cell biology of malaria. We also include more recent proteomic-based experiments in higher eukaryotes which are illuminating the fundamental role of CCT in regulating the TOR axis.

In yeast, analysis of the genetic network of CCT and its proteome revealed ∼300 genes/proteins which directly interact with the chaperonin. Notably, most components are not being actively folded by CCT, like actins and tubulins, but are cofactors and ancillary genetic effectors (Dekker et al., 2008; Willison, 2018b). CCT became coupled to many fundamental biological processes because it co-evolved with the cytoskeletal system in the Last Eukaryotic Common Ancestor (LECA) (Willison, 2018a). Independent computational analysis (Aswathy et al., 2016) and global experiments in yeast (Rizzolo et al., 2017) also show that CCT is highly selective towards the cytoskeleton in its network specificity. The yeast CCT ‘interactome’ was generated by mass spectrometric proteomics experiments using CCT-3CBP and CCT-6CBP tagged complexes and a Synthetic Genetic Array (SGA) screen for synthetic lethal interactions with a temperature-sensitive cct1-2 allele (Dekker et al., 2008) and subsequent analysis (Willison, 2018b).

### Abundance levels of CCT and clients

In 2012 the PaxDb proteomic database showed that CCT8 was the most abundant CCT subunit in yeast (247 ppm) and CCT1 the least abundant (95 ppm; Matalon et al., 2014) but in the current version (PaxDb online database; September 2022) CCT7 is the most abundant (341 ppm) and CCT2 the least (166 ppm). In the unified database of (Ho et al., 2018) CCT7 is the least abundant subunit (14,381 copies per cell) and CCT8 the most abundant (23,393 copies per cell). The variation in these three datasets is substantial: 2.5×, 2.05×, and 1.63× from highest to lowest. The covariance in the eight CCT subunit levels in haploid and diploid yeast is very tight compared to other multi-subunit complexes (Willison, 2018b) and CCT genes are the most haplo-insufficient in yeast along with ribosome subunits (Deutschbauer et al., 2005) so clearly their expression levels must be very tightly controlled. Willison estimates the abundance of CCT holo-complex in yeast at 3000 copies per haploid cell from protein purification yields (Willison, 2018b) which is considerably lower than the proteomics measurements would suggest, ∼15,000 copies per cell. Proteomic analysis records total protein levels and excludes CCT monomer and micro-complex abundances and are thus maximum estimates.

The unified yeast dataset (Ho et al., 2018) has actin (ACTI1) and tubulin (TUB1 and TUB2) levels at 134,173, 17,131 and 21,334 copies per cell, respectively (total 172,638 copies). Taking a cell division time of 90 mins in glucose at 30°C and G1/G2 phases at half cell cycle length (i.e., 45 mins) (Teufel et al., 2019) and assuming that half of the total pool is required by one bud (i.e., 172,638/2 = 86,319 proteins), one can estimate that 2.2% of the cytoskeletal protein, steady-state levels, requires folded every minute (86,319 proteins/45 mins = 1,918 which is 2.2% total). The CCT folding cycle takes 90 s at 30°C *in vitro* (McCormack et al., 2009) and therefore 2,877 complexes are committed to actin and tubulin folding at steady-state. Recently Kelly et al. (2022) 3x-FLAG tagged the endogenous CCT5 gene in human HEK293T cells and subjected 2.5 million CCT particles in the closed state to single particle cryo-EM analysis. They found 29.7% of the complexes were CCT-tubulin and 20% CCT-actin-PhLP2A. Half of the holo-CCT in this cell type is actively committed to folding the cytoskeletal proteins. Thus far, quantitation of the protein abundances in *Plasmodium* life cycle stages is not absolute, only relative, so it is not currently possible to estimate PfCCT levels.

### Post translational modifications

Thirty-five proteomic datasets (Swearingen and Lindner, 2018) generated from various *P. falciparum* life cycle stages show the occurrence of extensive post translational modifications (PTMs). Peptide-PTMs can be located interactively in the PlasmoDB online database (Release 59 August 2022). For example, there are 343 total peptides derived from the PfCCT5 subunit of which 54 are unique. Six MS/MS peptides were detected in PfCCT5 isolated from blood stage phospho- and total proteome analysis (Lasonder et al., 2012) and there are over 800 arginine methylated proteins in *Plasmodium* and all eight PfCCT subunits are arginine methylated (Zeeshan et al., 2017). These PTM mapping lists add an additional layer of analysis to couple to the CCT-proteome datasets in *P. falciparum* https://plasmodb.org/plasmo/app/record/gene/PF3D7_0320300#category:proteomics and yeast (https://www.yeastgenome.org/).

### Actin

**FIGURE 7:**
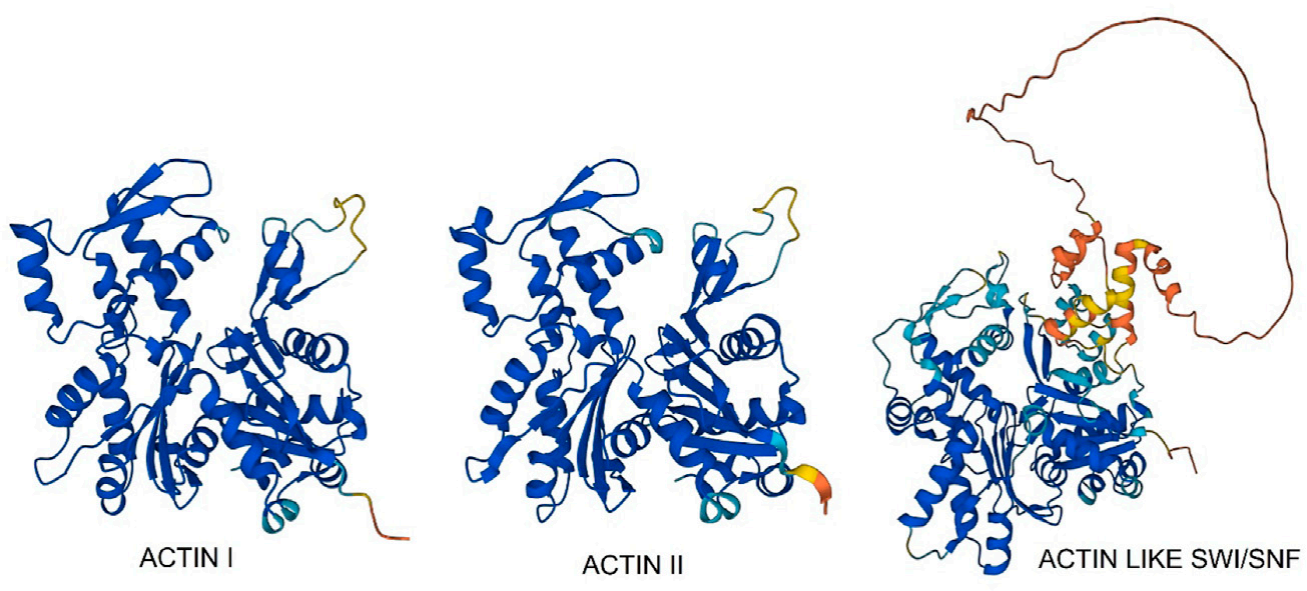
AlphaFold models of three *P. falciparum* actins found bound to PfCCT.

*Plasmodium* actins are among the most divergent known and the two major isoforms, PfAct1 and PfAct2 (Figure 7—Actin I and Actin II), are only 80% homologous. PfAct1 is expressed ubiquitously in all stages, but PfAct2 is restricted to the mosquito stages and transmittable sexual stages. PfAct2 can form long filaments, and disruption of its gene affects male gametocytes whereas PfAct1 is essential for viability. The crystallographic structure of PfAct1 shows lack of electron density for the C-terminus (Vahokoski et al., 2014), which is well-ordered in homologous actins and is essential for protein stability, interactions and filament formation. We found that *Plasmodium* actin is incompletely folded by heterologous protein-folding machinery and requires native *Plasmodium* CCT to fold (Olshina et al., 2016). The PfCCT proteome shows strong signal from PfAct1 and PfAct2 and two divergent actins (Figure 7; Table 1). One actin is related to eukaryotic nuclear actins involved in chromatin remodelling as a component of SW1/SNF complex (Figure 7). It has a large, natively disordered loop emanating from subdomain 2 which may be involved in condensate formation upon RNA binding in the nucleolus (Wiegand and Hyman, 2020). We screened all the PfCCT binding proteins for intrinsically disordered segments using the MobiDB-lite suite of IDP predictors. MobiDB-lite uses eight different predictors to derive a consensus which is then filtered for spurious short predictions. Consensus prediction is shown to outperform the single methods when annotating long ID regions (Necci et al., 2017).

### CCT cofactors—PLPs, prefoldins, Hsp40A

The phosducin-like proteins (PhLPs) are thioredoxin-fold, cofactors which enhance the binding of the actin, tubulin and Gβ protein substrates to CCT (Lukov et al., 2006; Willardson et al., 2007). In most eukaryotes, two different PLPs mediate actin and tubulin binding, essentially acting as specificity factors which enhance the respective substrate complexation with CCT. *In vivo* in *S. cerevisiae*, Plp1p and Plp2p are involved in tubulin and actin biogenesis and Plp2p, but not Plp1p, stimulates actin folding *in vitro* (McCormack et al., 2009). Genetic screens in yeast with temperature-sensitive mutants of Plp2p revealed several genes involved in G1/S cell cycle progression and the TOR pathway (Stirling et al., 2007). Plp2 mRNA synthesis is cell cycle-regulated allowing cell cycle-dependent actin folding.

**FIGURE 8:**
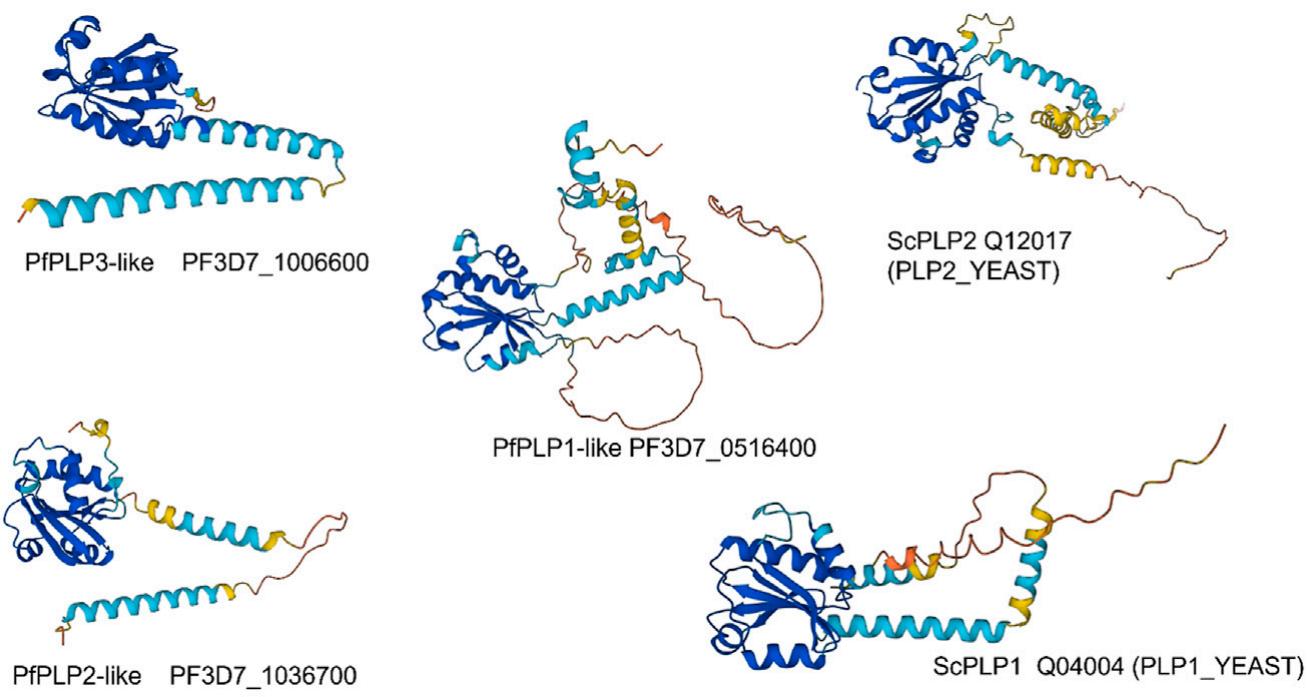
AlphaFold models of Phosducin-like proteins—*P. falciparum* v. *Saccharomyces cerevisiae*.

The PfCCT data finds abundant signal for three PhLPs (Table 1). PhLP2 in *Plasmodium berghei*, a rodent model of the malaria pathogen, can shuttle electrons between proteins in a thioredoxin-coupled redox assay *via* a highly reactive cysteine residue in the Trx domain and is suggested to regulate CCT folding activity (Kooistra et al., 2018). A comparison of AlphaFold models (Figure 8) of PfPhLPs (Varadi et al., 2022) and yeast PLP1 and PLP2 (McCormack et al., 2009) shows the conserved TWRC motif forms a non-canonical active site that is conserved across several *Plasmodium* species, including *P. falciparum*.

Five subunits of the hexameric prefoldin chaperone (Q8ILS7/subunit2:69.4) (Q8IBR6/subunit3:27.1) (Q8I3A4/subunit4:52.7) (Q8II82/subunit5:62.1) and (Q8I3Y6/subunit6:69.8), are detected in the proteome (the value after the colon is the coverage of the MS spectrum—see Supplementary Data S1). The AlphaFold model (Varadi et al., 2022) of subunit 5 shows a 40 amino acid natively disordered loop emanating from the linker between the two long helices. As with the unusual actin and WD40 proteins this region may be involved in coacervate formation in the erythrocyte cytosol.

There is a single Hsp40A chaperone (Q8IL88:41.5) and the Hsp70-Hsp90 organising protein (A0A144A2J9:14.5) and an activator of Hsp90 ATPase (O97244:10.6) in the dataset. Hsp40 interacts with actins and in the proteome of the yeast actin mutant strain (anc2) Hsp40 is found bound to CCT (see Figure 3F Dekker et al., 2008).

### WD40—Chromatin assembly, mRNA processing

**FIGURE 9:**
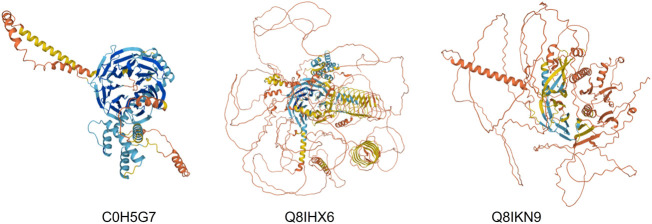
AlphaFold models of three *P. falciparum* WD40 propellor proteins bound to PfCCT.

**FIGURE 10:**
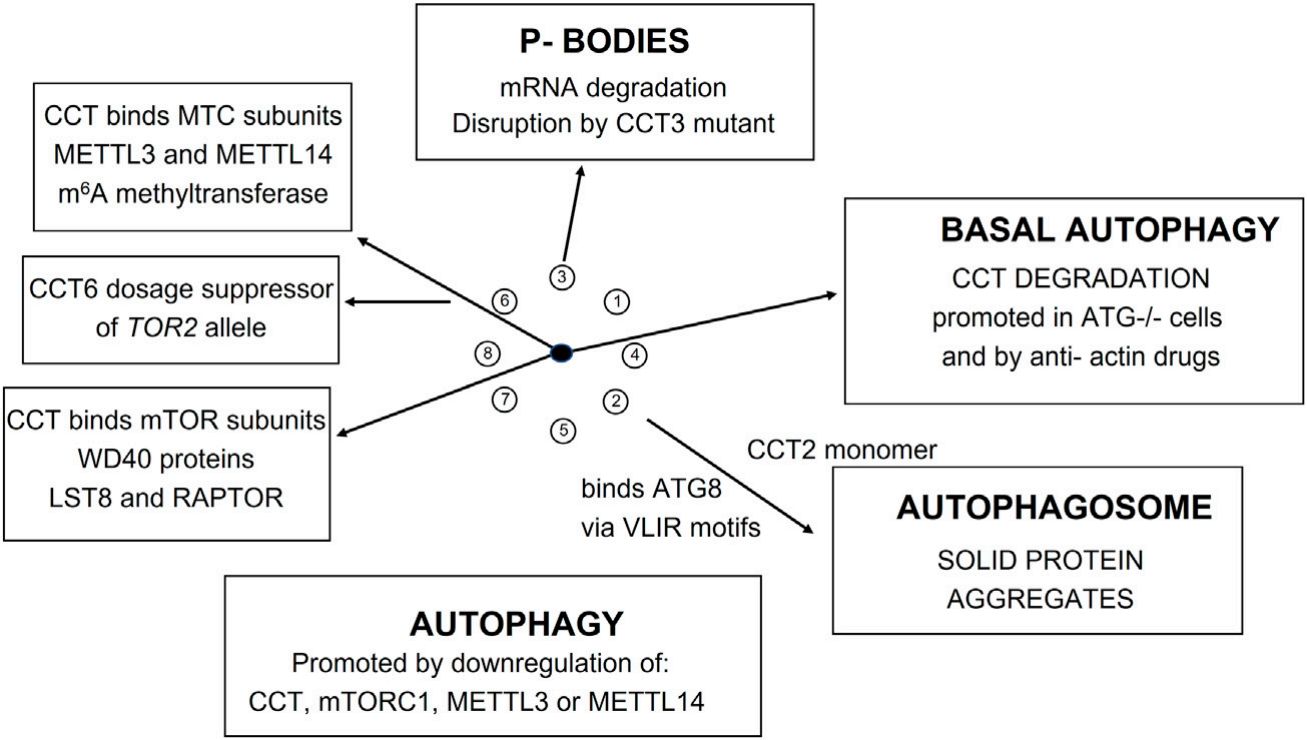
CCT involvement in mTOR pathway and autophagy.

CCT interacts with many WD40 proteins and has a core set of 30 members in yeast (Willison, 2018b). CCT binds the mTORC WD40 propellors, mLST8 and Raptor (see Figure 10). Cuéllar et al. (2019) obtained a 4.0 Å cryo-EM structure of a human mLST8-CCT intermediate, isolated directly from cells, which shows mLST8 in a near-native state bound to CCT deep within the folding chamber and interacting mainly with the disordered N- and C-termini of specific CCT subunits of both rings. Fourteen WD40 propellor repeat proteins are found in the *Plasmodium* CCT-precipitated proteome (Table 1). Some have clear orthologues in yeast such as the chromatin assembly factors and coatomer subunits, but others are novel and specific to *Plasmodium*. Again, there are huge loops of natively disordered structure in the AlphaFold models (examples shown in Figure 9) which suggests that CCT-WD40 complexes may act as nucleation structures for protein coacervates in the erythrocyte cytoplasm (Wiegand and Hyman, 2020).

### DNA—Transcription, chromatin assembly

Eight of the top 12 transcription factor hits are proteins with AP2 domains, again with huge loops of natively disordered structure in the AlphaFold models (data not shown). The yeast CCT proteome found extensive connectivity with transcriptional control and histone acetylation and deacetylation chemistry (Dekker et al., 2008).

### RNA—mRNA splicing, SSU and U3/U4 complexes

There are many interactions between CCT and RNA splicing and other RNA processing reactions in yeast (Dekker et al., 2008) and the same is found in the PfCCT-precipitated proteome (Table 1). There are six WD40 propellor proteins involved in RNA processing as in yeast (Willison, 2018b).

### Secretion—Coatomer I and II, trafficking particle complex

Upon invasion of the erythrocyte the malarial parasite secretes proteins into the surrounding cytoplasm to initiate formation of protein assemblies and vacuoles and membrane structures (Wickham et al., 2001). It is notable that WD40 propellor containing subunits of the Cop I and COP II secretion complexes are associated with PfCCT (Table 1) as are subunits of the trafficking protein particle complex, TRAPP (Q8I1Q2, C6KT27, Q8IE05).

### Autophagy and tor signaling

Protein network analyses in yeast (Dekker et al., 2008) and humans (Glatter et al., 2009) reveal direct interactions between CCT and several components of the mTOR (Mechanistic Target of Rapamycin Complex) signaling network and the PP2A protein phosphatase network. Yeast genetic analysis connected the CCT6 subunit of the chaperonin to TOR signalling many years ago (Schmidt et al., 1996) and directed mutagenesis of the ATPase activities of some of the chaperonin subunits perturbs mTOR activity at the transcriptional network level (Amit et al., 2010). Chemical genetic screens discovered impairment of yeast fitness to rapamycin in diploids hemizygous for CCT subunit genes (Hillenmeyer et al., 2008) and CCT acetylation and lifespan extension are perturbed in yeast SIR2 mutants (Liu et al., 2010) and the TOR axis is clearly involved in lifespan regulation in worms and flies. CCT is essential for organ growth in *Drosophila* by interacting with insulin/TOR signaling (Kim and Choi, 2019) and CCT8 and other subunits are phosphorylated following insulin stimulation (Kulkarni et al., 2016). mTORC1 regulates ribosome biogenesis *via* S6K and 4E-BP1 and the CCT2 subunit is a direct phosphorylation target of S6K kinase (Abe et al., 2009). The mammalian mTOR interacts with the β-propeller proteins, mLST8 and Raptor, to form the mTORC1 and mTORC2 complexes, respectively (Willison, 2018b). *Plasmodium* parasites do not possess a nutrient sensing TOR pathway and source all amino acids by degrading erythrocyte haemoglobin except isoleucine which is imported (Babbitt et al., 2012). Their rudimentary amino acid sensing pathway is mediated through initiation factor 2α (eIF2α). Several initiation factor-like subunits bind PfCCT including eIF2α.

The mTOR-CCT connection is under-appreciated because it is difficult to accommodate within conventional TOR signaling network models and experimental observations are often dismissed as the result of “non-specific” effects due to the extensive perturbations of the cytoskeleton by interfering with CCT mediated protein folding in the cell. Indeed (Date et al., 2022) show that disruption of actin dynamics in HeLa cells induces autophagic degradation of the CCT complex itself (Figure 10). Autophagy plays an important role in the intracellular protein quality control system by degrading abnormal organelles and proteins, including large protein complexes such as ribosomes.

mTORC1 has a critical role in RNA biogenesis and processing. Recently (Tang et al., 2021) found that the m^6^A methyltransferase complex (MTC) is a downstream effector of mTORC1 during autophagy in *Drosophila* and human cells. In order to characterize how mTORC1 regulates MTC (Tang et al., 2021) generated Protein-Protein Interaction Networks (PPINs) for *Drosophila* and human MTC by affinity purification and mass spectrometry (AP/MS) discovering 1,462 and 1,504 interactions, respectively, in the two datasets. N^6^-methyl-adenosine (m^6^A) is one of the most abundant chemical modifications in eukaryotic mRNA and is concentrated around stop codons and 3′UTRs. Specifically, mTORC1 regulates m^6^A methylation of ATG transcripts which induces their degradation and suppresses autophagy. Remarkably, the *Drosophila* CCT complex stabilizes the catalytic subunit of MTC, METTL3, and one of its partners, METTL14, required for RNA substrate recognition and efficient m^6^A modification. Depletion of either mTORC1, CCT, METTL3, or METTL14 compromises m^6^A RNA methylation and promotes autophagy. Importantly, the role of mTORC1-CCT-MTC signaling axis in regulating autophagy is conserved from *Drosophila* to mammals.

Atg8 is a unique ubiquitin-like protein that is covalently conjugated with a phosphatidylethanolamine through reactions similar to ubiquitination and plays essential roles in autophagy. Atg7 is the E1 enzyme for Atg8, and it activates the C-terminal Gly116 of Atg8 using ATP. We find two ATG7 related proteins in the PfCCT proteome (Q811A3 and Q8IC20). Taken together, these results indicate that mTORC1-regulated m6A methylation enhances degradation of specific Atg transcripts, which in turn inhibits autophagy. A recent study shows that the CCT2 monomer acts independently of the CCT holo-complex to promote autophagosome incorporation and clearance of protein aggregates with little liquidity *via* interacting with ATG8s and aggregation-prone proteins independent of cargo ubiquitination. Figure 10 shows a summary of the recent studies on the role of CCT in autophagic processes in flies and humans and P-body mediated mRNA degradation in yeast (Nadler-Holly et al., 2012).

### Protein translation—EF2-diphthamide modification pathway

In yeast, the elongation factor (EF2) is a strong interactor with CCT (Hit 16: Dekker et al., 2008). EF2 has a unique posttranslational modification of histidine. This modification is mediated by the diphthamide pathway and Dph1, the catalytic component of the multiprotein complex enzyme, was also found bound to CCT (Hit 30). PfCCT also binds PfEF2 (Q8IKW5:29.6) and a component of the diphthamide complex (A0A143ZY29:12.3). The diphthamide pathway is not essential for yeast growth but is involved in stress response and nutrient control. Interestingly, the iron/sulphur co-factor protein (Q8I5V5:22.3) required for the diphthamide reaction with histidine, is also found bound to CCT and contains a 7-bladed WD40 propellor (Table 1). Connecting partial data from two eukaryotic species indicates the utility of comparative proteomic studies in uncovering new roles for CCT in biochemical coupling of cytoskeletal protein folding flux to growth control processes.

The PfCCT genes have been identified as some of the most up-regulated genes in artemisinin (ART)-resistant parasites (Mok et al., 2015) and click chemistry-compatible activity-based probes incorporating the endoperoxide scaffold of ART identified CCT subunits and actin and tubulin as ART molecular target(s) in the asexual stages of the malaria parasite (Ismail et al., 2016). Recently, PfEF2 has been identified as the target of new anti-malarial tetrahydroquinoline compounds designed to overcome artemisinin resistance (Laleu et al., 2022) and it is possible that targeting PfCCT could be an effective anti-malarial strategy; hitting these CCT-bound enzyme complexes like E2F-dipthamide and autophagy signalling.

## Summary

Comparative eukaryotic proteomics has the potential to broaden our understanding of key cellular pathways, such as that involving the CCT complex, the focus of our study. Here we have presented a new proteome-derived interactome, which catalogues the protein substrates and regulators of the CCT complex from *P. falciparum*, a member of the divergent apicomplexan lineage of protozoan parasites that split many hundreds of millions of years ago from other eukaryotes. Despite the lineage divergence, many of the components of core pathways from the CCT interactome involved in cytoskeletal regulation and cell growth remain conserved in proteomes associated with CCT complexes from yeast to human to *Plasmodium*. This points to key areas of focus for generic understanding of the function and role of CCT as well as highlighting areas of study (core differences) that could provide targets for much needed antimalarials.

## Data Availability

The original contributions presented in the study are included in the article and in the Supplementary Material, further inquiries can be directed to the corresponding author.
